# Is FOLFOXIRI alone or combined with targeted therapy administered as first-line treatment a reasonable choice for most patients with mCRC? Systematic review and network meta-analysis

**DOI:** 10.18632/oncotarget.17725

**Published:** 2017-05-09

**Authors:** Mingyi Zhou, Ping Yu, Dengue Bilibili Hernick Davin, Yanrong Li, Yuanhe Wang, Lingyu Fu, Jingdong Zhang

**Affiliations:** ^1^ Department of Gynecology, Cancer Hospital of China Medical University, Liaoning Cancer Hospital & Institute, Shenyang 110042, Liaoning Province, PR China; ^2^ Department of Medical Oncology, The First Hospital of China Medical University, Shenyang 110001, Liaoning Province, PR China; ^3^ Department of Medical Oncology, Cancer Hospital of China Medical University, Liaoning Cancer Hospital & Institute, Shenyang 110042, Liaoning Province, PR China; ^4^ Department of Clinical Epidemiology and Evidence Based Medicine, The First Hospital of China Medical University, Shenyang 110001, Liaoning Province, PR China

**Keywords:** dose intensity, mCRC, prognosis, toxicities, network meta-anaylsis

## Abstract

Whether the intensive administration of folinic acid, 5-fluorouracil, oxaliplatin and irinotecan (FOLFOXIRI) alone or combined with target therapy as first-line treatment could improve the prognosis of metastatic colorectal cancer (mCRC) patients is controversial. PubMed, the Cochrane Collaboration Central Register of Controlled Clinical Trials, Cochrane Systematic Reviews, ClinicalTrials.gov, the databases of conferences were queried to identify RCTs evaluating the efficacies and toxicities of intensive therapies used for first-line treatment of mCRC patients. The search included articles dated from the inception of these resources until March 31, 2017. We estimated HRs for OS and PFS and RRs for ORR, the R0 resection rate, and toxicities. Ten RCTs comprising 2,506 patients were included in this network meta-analysis. The PFS of patients administered FOLFOXIRI plus target therapy experienced prolonged PFS and OS and improved ORRs compared with FOLFOX/FOLFIRI plus target therapy (PFS: HR 0.71, 95% CI 0.59–0.86; OS: HR 0.81, 95% CI 0.69–0.94; ORR: RR 1.66, 95% CI 0.96–2.88; R0 resection rate: RR 2.66, 95% CI 1.86–3.82). There were no significant differences between PFS, OS, ORRs, or R0 resection rates and toxicities of patients administered FOLFOXIRI and FOLFOX/FOLFIRI plus target therapy. Further, FOLFOXIRI plus target therapy did not increase toxicities compared with FOLFOX/FOLFIRI plus target therapy. FOLFOXIRI plus target therapy when administered as first-line treatment of patients with mCRC is the best choice and did not increase toxicities. The patients with *RAS/BRAF* mutations could benefit from FOLFOXIRI plus Bev. FOLFOXIRI is as effective as FOLFOX/FOLFIRI plus target therapy.

## INTRODUCTION

First-line treatment using folinic acid, fluorouracil, and oxaliplatin (FOLFOX) or folinic acid, 5-fluorouracil, and irinotecan (FOLFIRI) combined with target therapy prolongs the overall survival (OS) of patients with metastatic colorectal cancer (mCRC) patients compared with dual chemotherapy, particularly when administered to patients without *RAS* or *BRAF* mutations [1–7]. A study that used a regression model to analyze the correlation between the percentages of patients treated with triplet chemotherapy and the outcomes, found that OS is prolonged [8]. However, this model was derived using the data of a single arm of a study of patients with mCRC who were administered chemotherapy as first-line treatment. Moreover, the dose intensities of chemotherapy and targeted therapy were not considered.

Randomized controlled trials (RCTs) compared the efficacy and toxicities of triple chemotherapy (folinic acid, 5-fluorouracil, oxaliplatin and irinotecan [FOLFOXIRI]) plus target therapy with those of triple or double chemotherapy (FOLFOX or FOLFIRI) plus target therapy [9–11]. The efficacies and toxicities of triple and double chemotherapy were compared as well. However, whether dose intensity is the most important prognostic factor is controversial. We asked therefore whether FOLFOXIRI plus target therapy administered as first-line treatment improves the prognosis of patients with mCRC and if there is a significant difference in efficacy between FOLFOXIRI and double chemotherapy plus target therapy?

## RESULTS

**Figure 1:**
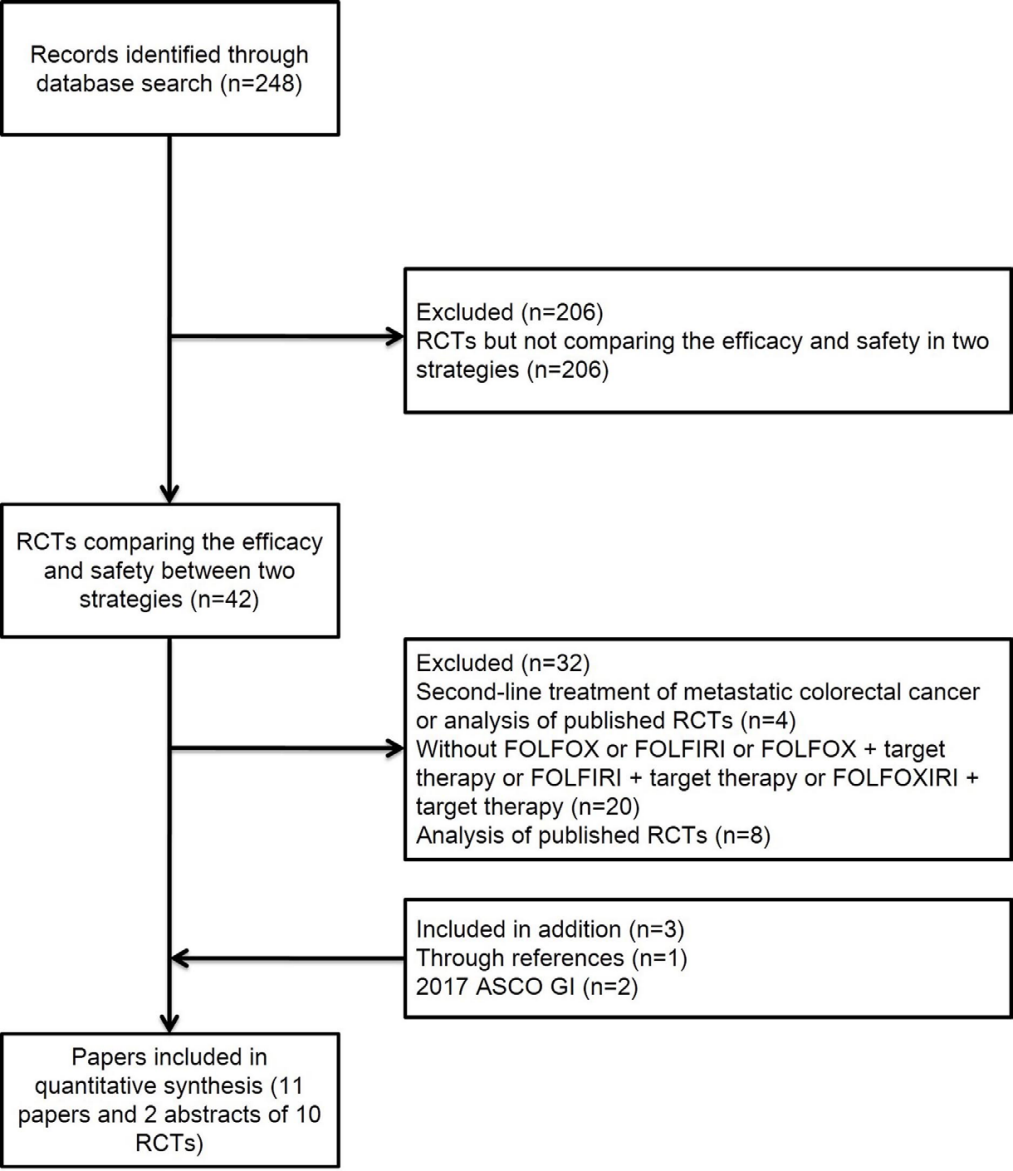
Literature search and selection of studies Abbreviation: RCT, randomized controlled trial.

The titles and abstracts of 248 studies were reviewed. After the initial screen, we performed a detailed assessment of potentially eligible papers and selected 11 papers and 2 abstracts of 10 randomized controlled trials (RCT) [1–7, 9–14] (Figure 1). FIRE3 study [15], PEAK study [16] and CALGB 80405 study [17] were not included because the regimens both in the experimental groups and the control groups were recognized as FOLFOX or FOLFIRI plus target therapy in our network meta-analysis. And the survival of these three studies were consistent with the studies included. Table 1 shows the characteristics of these 10 RCTs. The patients with mCRC (n = 2,506) were randomized into treatment vs control groups (Table 1). Methodological quality assessment was performed according to the latest guidelines in the Cochrane Handbook for Systematic Reviews of Interventions. The quality of each included study was high (Table 2).

**Table 1:** Characteristics of the included randomized control trials

Study	Study design	Treatment schedule
OPUS 2011, 2015	FOLFOX4+Cetux.	Cetux.: initial dose 400 mg/m^2^ and 250 mg/m^2^/week thereafter, Q2W. FOLFOX4: oxaliplatin 85 mg/m^2^; folinic acid 200 mg/m^2^; 5-FU 400 mg/m^2^ IV bolus and 600 mg/m^2^ 22-hour continuous infusion on days 1 and 2, Q2W.
	FOLFOX4	FOLFOX4: oxaliplatin 85 mg/m^2^; folinic acid 200 mg/m^2^; 5-FU 400 mg/m^2^ IV bolus and 600 mg/m^2^ 22-hour continuous infusion on days 1 and 2, Q2W.
CRYSTAL 2011, 2015	FOLFIRI+Cetux.	Cetux.: initial dose 400 mg/m^2^ and 250 mg/m^2^/week thereafter, followed after 1 hour by FOLFIRI, Q2W. FOLFIRI: irinotecan 180 mg/m^2^, day 1, infused over 30–90 minutes; leucovorin 200 mg/m^2^ L-form, or 400 mg/m^2^ racemic, infused over 2 hours; fluorouracil 400 mg/m^2^ IV bolus and 2400 mg/m^2^ 46-hour continuous infusion, Q2W.
	FOLFIRI	FOLFIRI: irinotecan 180 mg/m^2^, on day 1, infused over 30–90 minutes; leucovorin 200 mg/m^2^ L-form, or 400 mg/m^2^ racemic, infused over 2 hours; fluorouracil 400 mg/m^2^ IV bolus and 2400 mg/m^2^ 46-hour continuous infusion, Q2W.
PRIME 2010, 2013	FOLFOX4+Panit.	Panit.: IV over 1 hour, 6 mg/kg on day 1 before FOLFOX4, Q2W FOLFOX4: oxaliplatin 85 mg/m^2^ IV infusion on day 1; leucovorin 200 mg/m^2^ IV infusion; fluorouracil 400 mg/m^2^ IV bolus and 600 mg/m^2^ 22-hour continuous infusion on days 1 and 2, Q2W.
	FOLFOX4	FOLFOX4: oxaliplatin 85 mg/m^2^ IV infusion on day 1; leucovorin 200 mg/m^2^ IV infusion; fluorouracil 400 mg/m^2^ IV bolus and 600 mg/m^2^ 22-hour continuous infusion on days 1 and 2, Q2W.
TRIBE 2015	FOLFOXIRI+bevacizumab.	Bevacizumab: 5 mg/kg, Q2W. FOLFOXIRI: irinotecan 165 mg/m^2^ intravenous infusion for 60 min, followed by an 85 mg/m^2^ intravenous infusion of oxaliplatin given concurrently with leucovorin at a dose of 200 mg/m^2^ for 120 min, followed by a 3200 mg/m^2^ continuous infusion of fluorouracil for 48 h.
	FOLFIRI+bevacizumab.	Bevacizumab: 5 mg/kg, Q2W. FOLFIRI: irinotecan 180 mg/m^2^ intravenous infusion for 60 min followed by a 200 mg/m^2^ intra venous infusion of leucovorin for 120 min, a 400 mg/m^2^ intravenous bolus of fluorouracil, and a 2400 mg/m^2^ continuous infusion of fluorouracil for 46h.
OLIVIA 2015	FOLFOXIRI+bevacizumab.	Bevacizumab: 5 mg/kg, Q2W. FOLFOXIRI: oxaliplatin 85 mg/m^2^, irinotecan 165 mg/m^2^, folinic acid 200 mg/m^2^, 5-fluorouracil 3200 mg/m^2^ (46-h infusion)
	mFOLFOX-6+bevacizumab.	Bevacizumab: 5 mg/kg, Q2W. mFOLFOX-6: oxaliplatin 85 mg/m^2^, folinic acid 400 mg/m^2^, 5-fluorouracil 400 mg/m^2^ (bolus) then 2400 mg/m^2^ (46-h infusion)
AVF2107 2009	IFL+bevacizumab.	Bevacizumab IFL: irinotecan, leucovorin, and leucovorin
	IFL+placebo.	Placebo IFL: irinotecan, leucovorin, and leucovorin
GONO 2007	FOLFOXIRI	Irinotecan 165 mg/m^2^ on day 1; Oxaliplatin 85 mg/m^2^ on day 1; Leucovorin 200 mg/m^2^ on day 1; 5-fluorouracil 3200 mg/m^2^ 48-hour continuous infusion starting on day 1. Repeated every 2 weeks.
	FOLFIRI	Irinotecan 180 mg/m^2^ on day 1; Leucovorin 100 mg/m^2^ on day 1 and day 2; 5-fluorouracil 400 mg/m^2^ bolus followed by 5-fluorouracil 600 mg/m^2^ 22-hour continuous infusion on day 1 and day 2. Repeated every 2 weeks.
HORG 2006	FOLFOXIRI	CPT-11 at the dose of 150 mg/m^2^ as a 30 min IV infusion on day 1; LV was given at the dose of 200 mg/m^2^ as a 2 h IV infusion, followed by 5FU 400 mg/m^2^ as IV bolus, and then, 600 mg/m^2^ as a 22 h continuous IV infusion, on days 2 and 3; L-OHP was administered on day 2 at the dose of 65 mg/m^2^ as a 2 h IV infusion in parallel with LV but using different lines.
	FOLFIRI	CPT-11 at the dose of 180 mg/m^2^ as a 30 min IV infusion on day 1; LV was given at the dose of 200 mg/m^2^ as a 2 h IV infusion, followed by 5FU 400 mg/m^2^ as IV bolus, and then, 600 mg/m^2^ as a 22 h continuous IV infusion, on days 1 and 2.
STEAM 2017	FOLFOXIRI + bevacizumab	Bevacizumab: 5 mg/kg Q2W. FOLFOXIRI Q4W in a 4–6 month induction phase, followed by bevacizumab-containing maintenance treatment
	FOLFOX + bevacizumab	Bevacizumab: 5 mg/kg Q2W. FOLFOX Q3W in a 4–6 month induction phase, followed by bevacizumab-containing maintenance treatment
CHARTA 2017	FOLFOXIRI + bevacizumab	Bevacizumab: 5 mg/kg, Q2W. FOLFOXIRI: irinotecan 165 mg/m^2^ intravenous infusion for 60 min, followed by an 85 mg/m^2^ intravenous infusion of oxaliplatin given concurrently with leucovorin at a dose of 200 mg/m^2^ for 120 min, followed by a 3200 mg/m^2^ continuous infusion of fluorouracil for 48 h.
	FOLFOX + bevacizumab	Bevacizumab: 5 mg/kg Q2W. FOLFOX Q3W in a 4–6 month induction phase, followed by bevacizumab-containing maintenance treatment

Abbreviations: Q2W, every 2 weeks; IV, intravenous.

**Table 2:** Methodological quality of included RCTs

study	Sequence generation	Allocation sequence concealment	Blinding of participants and personnel	Blinding of outcome assessment	Incomplete outcome data	Selective outcome reporting	Other risk of bias
OPUS 2011	adequate	adequate	Not report	yes	no	no	no
OPUS 2015	adequate	adequate	Not report	yes	no	no	no
CRYSTAL 2011	adequate	adequate	Not report	yes	no	no	no
CRYSTAL 2015	adequate	adequate	Not report	yes	no	no	no
PRIME 2010	adequate	adequate	Not report	yes	no	no	no
PRIME 2013	adequate	adequate	Not report	yes	no	no	no
TRIBE 2015	adequate	adequate	Not report	yes	no	no	no
OLIVIA 2015	adequate	adequate	Not report	yes	no	no	no
AVF2107	adequate	adequate	Not report	yes	no	no	no
GONO 2007	adequate	adequate	Not report	yes	no	no	no
HORG 2006	adequate	adequate	Not report	yes	no	no	no
STEAM 2017	adequate	adequate	Not report	yes	no	no	no
CHARTA 2017	adequate	adequate	Not report	yes	no	no	no

ITT, intention to treat. Unclear reporting of allocation was considered inadequate.

**Figure 2:**
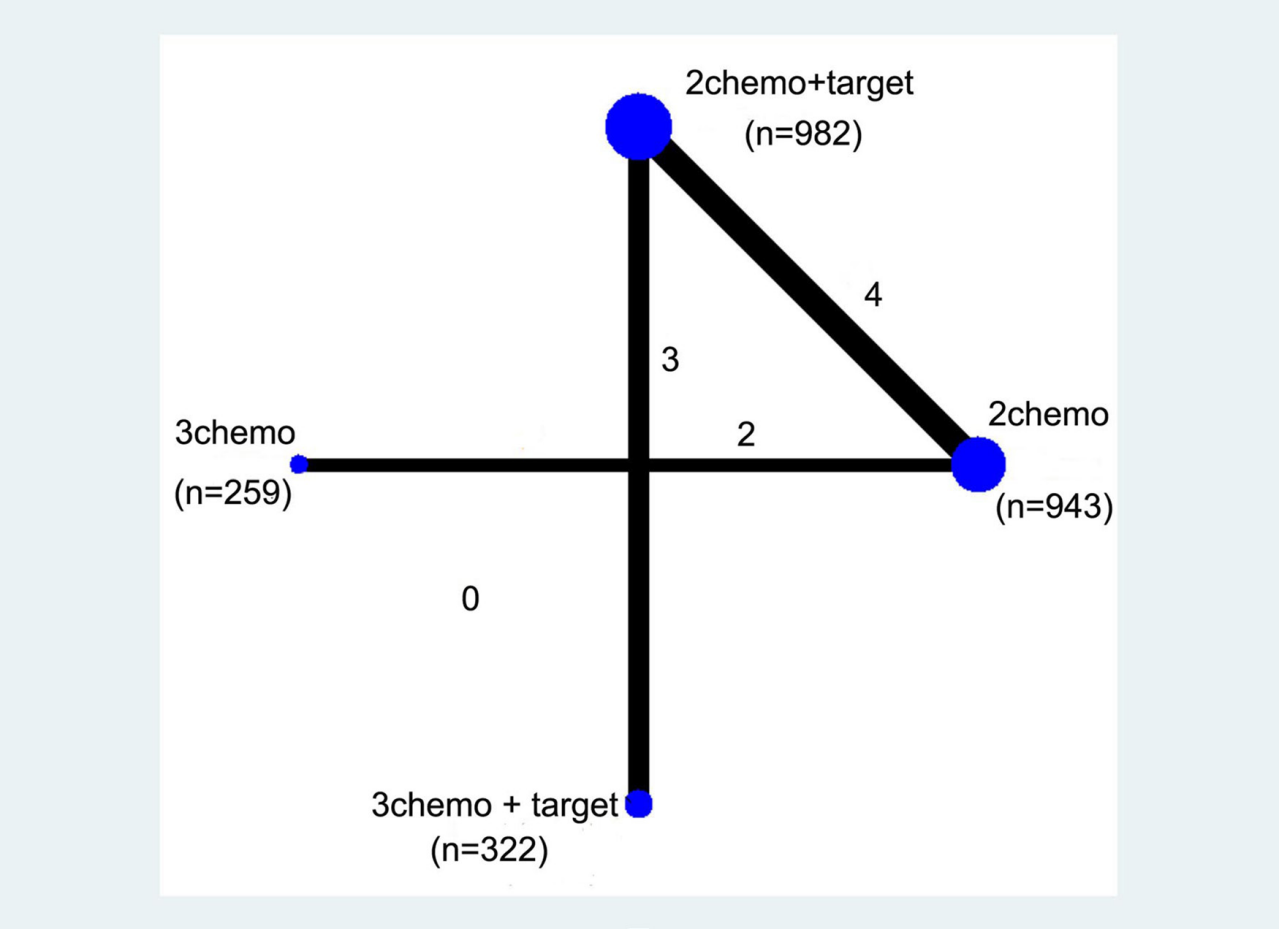
Network of the comparisons included in the network meta-analysis The sizes of the nodes are proportional to the numbers of patients (in parentheses) randomized to receive the treatment. The width of the lines is proportional to the number of trials (next to the line) comparing the connected treatments.

The network of the comparisons is shown in Figure 2. Four regimens from 10 trails were included in the network. Four studies compared double chemotherapy plus target therapy with double chemotherapy. Four studies compared double chemotherapy plus target therapy with triple chemotherapy plus target therapy. Two studies compared triple chemotherapy with double chemotherapy.

**Figure 3:**
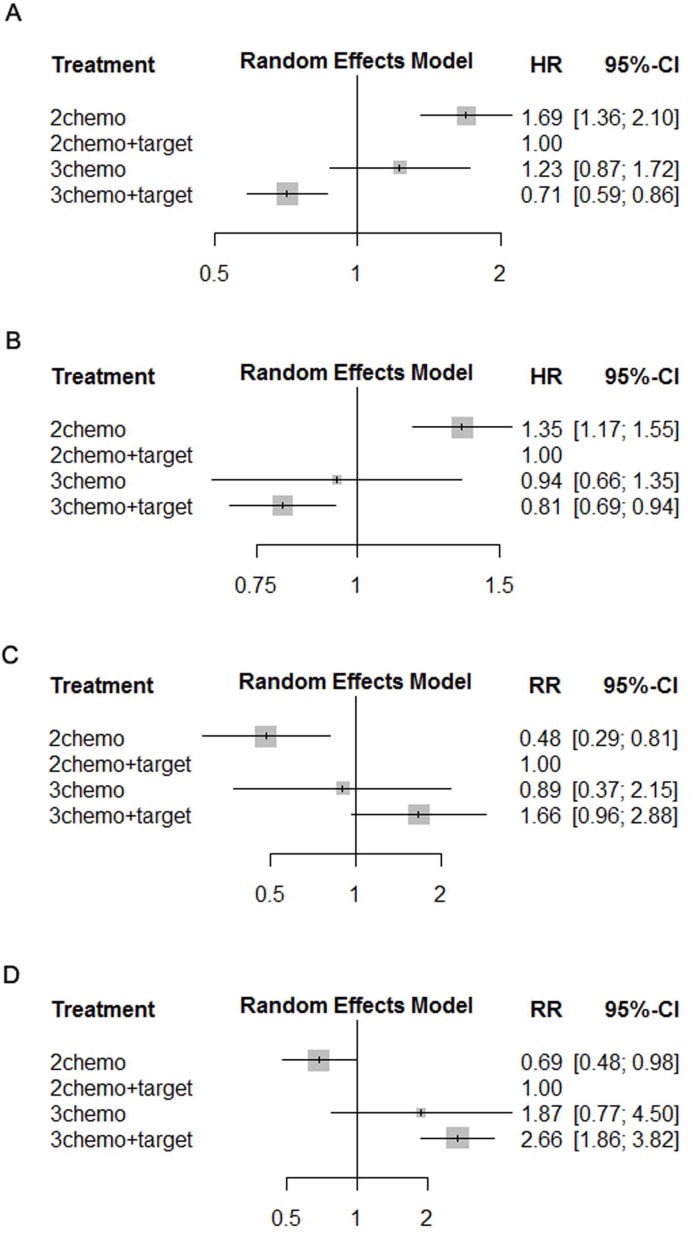
Pooled HRs of PFS **(A)** and OS **(B)**, pooled RRs of ORR **(C)**, and R0-resection rate **(D)** determined using network meta-analysis. Abbreviations: HR, hazard ratio, PFS, progression-free survival; OS, overall survival; ORR, objective response rate.

The pooled HRs of PFS of patients administered an individual regimen compared with FOLFOX or FOLFIRI plus target therapy in the network meta-analysis shows that the PFS of patients administered FOLFOXIRI plus target therapy was longer compared with that of FOLFOX or FOLFIRI plus target therapy (HR 0.71, 95% CI 0.59–0.86) (Figure 3A). There was no difference between FOLFOXIRI and FOLFOX or FOLFIRI plus target therapy (HR 1.23, 95% CI 0.87–1.72) (Figure 3A). Pooled HRs of OS of patients administered individual regimens compared with FOLFOX or FOLFIRI plus target therapy in the network meta-analysis shows that the OS of patients for FOLFOXIRI plus target therapy was longer compared with that of FOLFOX or FOLFIRI plus target therapy (HR 0.81, 95% CI 0.69–0.94) (Figure 3B). There was no difference between FOLFOXIRI and FOLFOX or FOLFIRI plus target therapy (HR 0.94, 95% CI 0.66–1.35) (Figure 3B).

The pooled RRs of ORR of patients administered individual regimens compared with FOLFOX or FOLFIRI plus target therapy in the network meta-analysis shows that the ORR of patients administered FOLFOXIRI plus target therapy was higher compared with that of FOLFOX or FOLFIRI plus target therapy (RR 1.66, 95% CI 0.96–2.88) (Figure 3C). There was no difference between FOLFOXIRI and FOLFOX or FOLFIRI plus target therapy (RR 0.89, 95% CI 0.37–2.15) (Figure 3C). The pooled RRs of the R0 resection rate of patients administered individual regimens compared with FOLFOX or FOLFIRI plus target therapy in the network meta-analysis shows that the R0 resection rate of patients administered FOLFOXIRI plus target therapy was higher compared with that of FOLFOX or FOLFIRI plus target therapy (RR 2.66, 95% CI 1.86–3.82) (Figure 3D). There was no significant difference between FOLFOXIRI and FOLFOX or FOLFIRI plus target therapy (RR 1.87, 95% CI 0.77–4.50) (Figure 3D).

**Figure 4:**
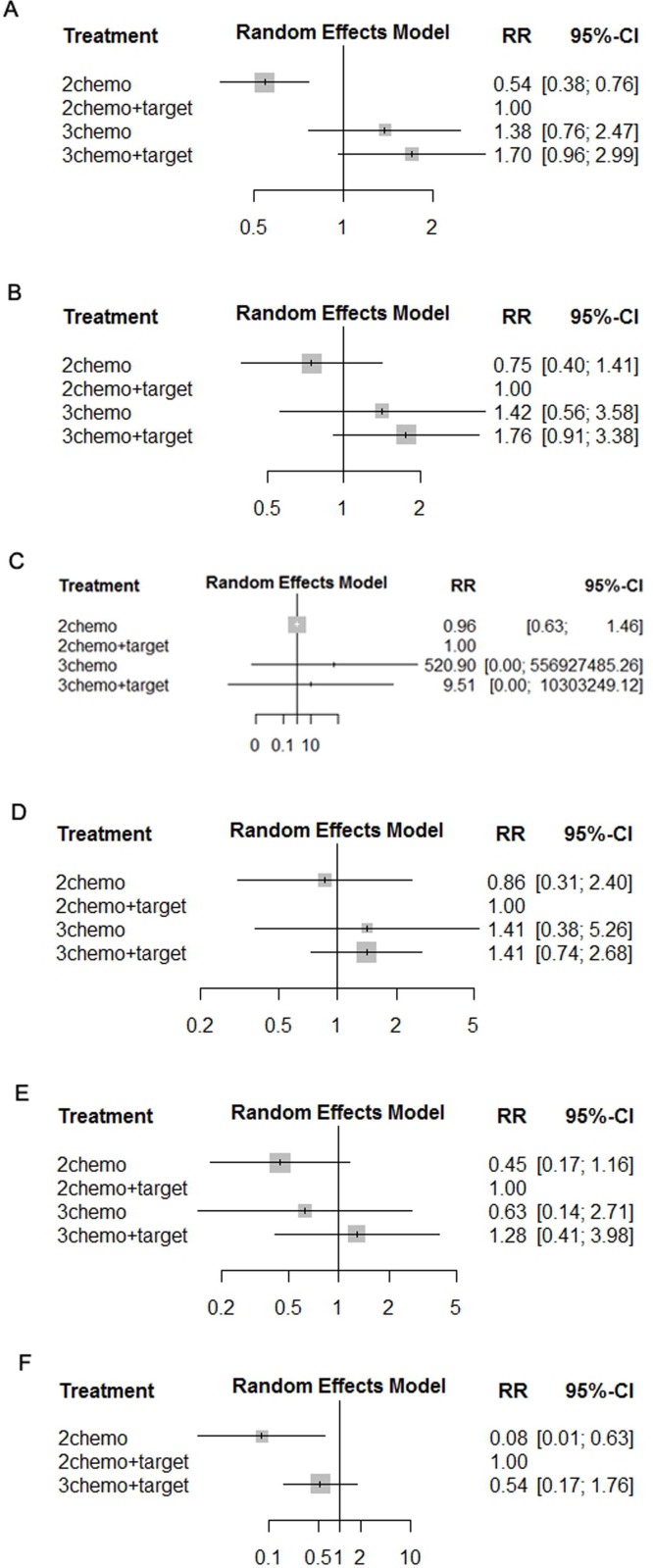
Pooled RRs of toxicities determined using network meta-analysis Diarrhea **(A)**, Neutropenia **(B)**, Neurologic toxicities **(C)**, Febrile neutropenia **(D)**, Fatigue **(E)**, and Venous thrombosis **(F)**.

The pooled RRs of toxicities of patients administered individual regimens compared with double chemotherapy plus targeted therapy in the network meta-analysis shows no increase in toxicities between FOLFOXIRI and FOLFOX or FOLFIRI plus target therapy (Figure 4). Further, FOLFOXIRI plus target therapy did not increase toxicities compared with FOLFOX or FOLFIRI plus target therapy (Figure 4B).

We also performed the subgroup analysis for the patients with *RAS/BRAF* mutations and the patients without *RAS* mutations. For the patients with *RAS* mutations (n = 454), FOLFOXIRI plus Bevacizumab (Bev) significantly prolonged the PFS compared with FOLFOX or FOLFIRI plus Bev (HR 0.75, 95% CI 0.62–0.92). For the patients with *BRAF* mutations (n = 54), FOLFOXIRI plus Bev prolonged the PFS compared with FOLFOX or FOLFIRI plus Bev, but the difference was not significant (HR 0.64, 95% CI 0.36–1.15). For the patients without *RAS* mutations (n = 292), FOLFOXIRI plus Bev significantly prolonged the PFS compared with FOLFOX or FOLFIRI plus Bev (HR 0.73, 95% CI 0.57–0.94) (Supplementary Figure 1).

## DISCUSSION

The results of this network meta-analysis show that first-line treatment using FOLFOXIRI plus target therapy prolonged PFS and OS and improved the ORR and R0 resection rate of patients with mCRC without a significant increase in toxicities. The patients with *RAS/BRAF* mutations could benefit from FOLFOXIRI plus Bev compared with FOLFOX or FOLFIRI plus Bev. Triple chemotherapy used in some studies was as effective as double chemotherapy plus target therapy and was associated with acceptable toxicities. Therefore, patients who cannot afford target therapy may have the option of choosing FOLFOXIRI as their first-line treatment.

This study is the first to our knowledge to conduct a network meta-analysis that ranks the efficacy and toxicities of intensive therapy strategies. NCCN 2017 version 2 guidelines recommend a choice of FOLFOX, FOLFIRI, CapeOx, infusion 5-FU/LV or capecitabine, or FOLFOXIRI, with or without targeted agents as initial therapy for mCRC patients appropriate for intensive therapy. But which one is the best choice was still controversial. The 2016 ESMO guidelines recommend that the goal for treating patients with potentially resectable or nonresectable mCRC who have the ability to withstand intensive therapy should achieve shrinkage of clinically relevant tumors to the maximum extent possible [18]. Further, the 2016 ESMO guidelines state that for patients who are candidates for therapies that maximize tumor shrinkage or for those with *BRAF* mutations, the cytotoxic triplet FOLFOXIRI plus Bev is an option. But the grade of this evidence was IIB [18].

Considering the absence of sufficient evidence, the application of intensive therapeutic strategies for treating patients with mCRC is limited. The results of our network meta-analysis suggest that patients with mCRC may benefit from FOLFOXIRI plus target therapy as first-line treatment. Therefore, FOLFOXIRI or FOLFOXIRI plus Bev may be a more suitable option for patients able to withstand intensive treatment. Moreover, the analysis of toxicities presented here show that FOLFOXIRI did not significantly increase toxicities compared with those associated with FOLFOX or FOLFIRI plus target therapy. And FOLFOXIRI plus target therapy also did not increase toxicities compared with those associated with FOLFOX or FOLFIRI plus target therapy, except for neutropenia.

The number of patients with mCRC who can afford target treatment is limited, and the efficacies and toxicities of triple chemotherapy and double chemotherapy plus target chemotherapy were not determined by RCTs. The results of our indirect comparison using network meta-analysis suggest that patients treated with FOLFOXIRI achieved similar PFS, OS, ORR, and R0 resection rates compared with patients treated with double chemotherapy plus target chemotherapy. Therefore, patients with mCRC who cannot afford target therapy may choose FOLFOXIRI as their first-line treatment.

This study did not analyze patients’ ages or their performance status because only some certain criteria for the patients administered FOLFOXIRI with or without target therapy were referred in the included studies but not consistent. For example, in the STEAM study [11], patients aged 18–75 years were required to meet Eastern Cooperative Oncology Group (ECOG) performance status ≤1. The inclusion criteria of the OLIVIA [10], TRIBE [9], and GONO [12] studies were age ≥18 years and ECOG performance status ≤1; age 18–70 years, ECOG performance status ≤2; and age 71–75-years, ECOG performance status = 0; respectively. In the HORG study [13], which did not impose an age limit, 56% of patients treated with FOLFOXIRI were aged ≥65 years.

Our network meta-analysis has certain limitations. First, target therapy has been used as first-line treatment for mCRC since 2004. Thus, target therapy was not administered to study and control groups in the GONO study [12], which may have biased the present data. Second, testing for *RAS* and *BRAF* mutations is not currently required for treatment using Bev, and the HRs of all patients without data for *RAS* mutational status included in the studies were administered bevacizumab but not an anti-EGFR antibody. Third, the number of eligible studies was small. Therefore, future RCTs must include larger numbers of subjects.

We conclude that FOLFOXIRI plus target therapy administered as first-line treatment is the best choice for patients with mCRC, because it is not significantly associated with increased toxicities. The patients with *RAS/BRAF* mutations could benefit from FOLFOXIRI plus Bev. Further, FOLFOXIRI is as effective as double chemotherapy plus target therapy.

## MATERIALS AND METHODS

### Search strategy

This network meta-analysis was performed according to the Preferred Reporting Items for Systematic Reviews and Meta-Analyses (PRISMA) guidelines [19]. We searched the abstracts of RCTs to evaluate the efficacies and toxicities of intensive therapies administered as first-line treatment of patients with mCRC. The resources searched included PubMed, the Cochrane Collaboration Central Register of Controlled Clinical Trials, Cochrane Systematic Reviews, ClinicalTrials.gov, and the databases of the European Society for Medical Oncology and American Society of Clinical. The search included articles dated from the inception of these resources until March 31, 2017 (the list of search terms is included in the Supplementary Data). We reviewed the bibliographies of these reports as well as related reviews to identify articles. These papers were subjected to manual searches. Our network meta-analysis included studies that compared different intensive therapies.

### Quality assessment and data extraction

Two investigators (Z-MY, Y-P) independently reviewed the entire text of eligible studies. Information was extracted and inserted into an electronic database that included patient characteristics, inclusion and exclusion criteria, treatment protocols, and outcomes. Any disagreement between reviewers was discussed with the other coauthors until a consensus was reached.

### Data synthesis and analysis

Outcomes of this study included progression-free survival (PFS), OS, ORR, and the R0 resection rate. Random-effects models were used to account for the heterogeneity among studies. Standard meta-analysis was performed using Stata 12.1 (StataCorp, College Station, TX, USA). Network meta-analysis was performed using a netmeta package developed according to the theories of a classical frequentist setting included in the R language framework.

### SUPPLEMENTARY MATERIALS FIGURE


