# Supplementary material for: Is FOLFOXIRI alone or combined with targeted therapy administered as first-line treatment a reasonable choice for most patients with mCRC? Systematic review and network meta-analysis
Source: Oncotarget. 2017 May 9;8(37):62339–48. doi: 10.18632/oncotarget.17725 (PMC5617509; doi:10.18632/oncotarget.17725)
Supplement: Supplementary file 1 [file oncotarget-08-62339-s001.pdf]

Is FOLFOXIRI alone or combined with targeted therapy administered as first-line treatment a reasonable choice for most patients with mCRC? Systematic review and network meta-analysis

SUPPLEMENTARY MATERIALS

APPENDIX

Search strategy

Pubmed:

("metastatic colorectal cancer" [Title/Abstract] OR "Colorectal Neoplasms" [MeSH Terms] OR "Colonic Neoplasms" [MeSH Terms] OR "Sigmoid Neoplasms" [MeSH Terms] OR "Rectal Neoplasms" [MeSH Terms]) AND (Humans [MeSH Terms] OR Adult [MeSH Terms] OR Aged [MeSH Terms] OR "Middle Aged" [MeSH Terms] OR "Young Adult" [MeSH Terms] OR Male [MeSH Terms] OR Female [MeSH Terms]) AND ("Organoplatinum Compounds" [MeSH Terms] OR chemotherapy* [Title/Abstract] OR Fluorouracil [MeSH Terms] OR Capecitabine [Title/Abstract] OR Tegafur [Title/Abstract] OR Leucovorin [Title/Abstract] OR Irinotecan [Title/Abstract] OR "Antineoplastic Combined Chemotherapy Protocols" [MeSH Terms] OR "Angiogenesis Inhibitors" [MeSH Terms] "Receptor, Epidermal Growth Factor" [MeSH Terms] OR "Antibodies, Monoclonal" [MeSH Terms] OR "Antibodies, Monoclonal, Humanized" [MeSH Terms] OR "Antibodies, Monoclonal, Murine-Derived" [MeSH Terms] OR "Molecular Targeted Therapy" [MeSH Terms] OR bevacizumab [Title/Abstract] OR cetuximab [Title/Abstract] OR panitumumab [Title/Abstract] OR ramucirumab [Title/Abstract] OR matuzumab [Title/Abstract] OR aflibercept [Title/Abstract]) AND ("Follow-Up Studies" [MeSH Terms] OR "Treatment Outcome" [MeSH Terms] OR "Prognosis" [MeSH Terms] OR "Drug-Related Side Effects and Adverse Reactions" [MeSH Terms] OR "Side Effect*" [Title/Abstract] OR "Adverse Drug Reaction*" [Title/Abstract] OR "toxicit*" [Title/Abstract]) AND ("Randomized Controlled Trial" [Publication Type] NOT "Clinical Trial, Phase I" [Publication Type]).

Cochrane Library:

http://onlinelibrary.wiley.com/cochranelibrary/search/advanced?hiddenFields.strategySortBy=last-modified-date;desc&hiddenFields.showStrategies=false&hiddenFields.containerId=9122339467944699691&hiddenFields.originalContainerId=&hiddenFields.etag=7574179336855920768&meshOrBasicAppended=true#

American Society of Clinical Oncology database of abstracts:

Metastatic colorectal cancer (http://meetinglibrary.asco.org/abstracts).

ClinicalTrials.gov:

Category: "Carcinoma, Colorectal" (http://clinicaltrials.gov/).

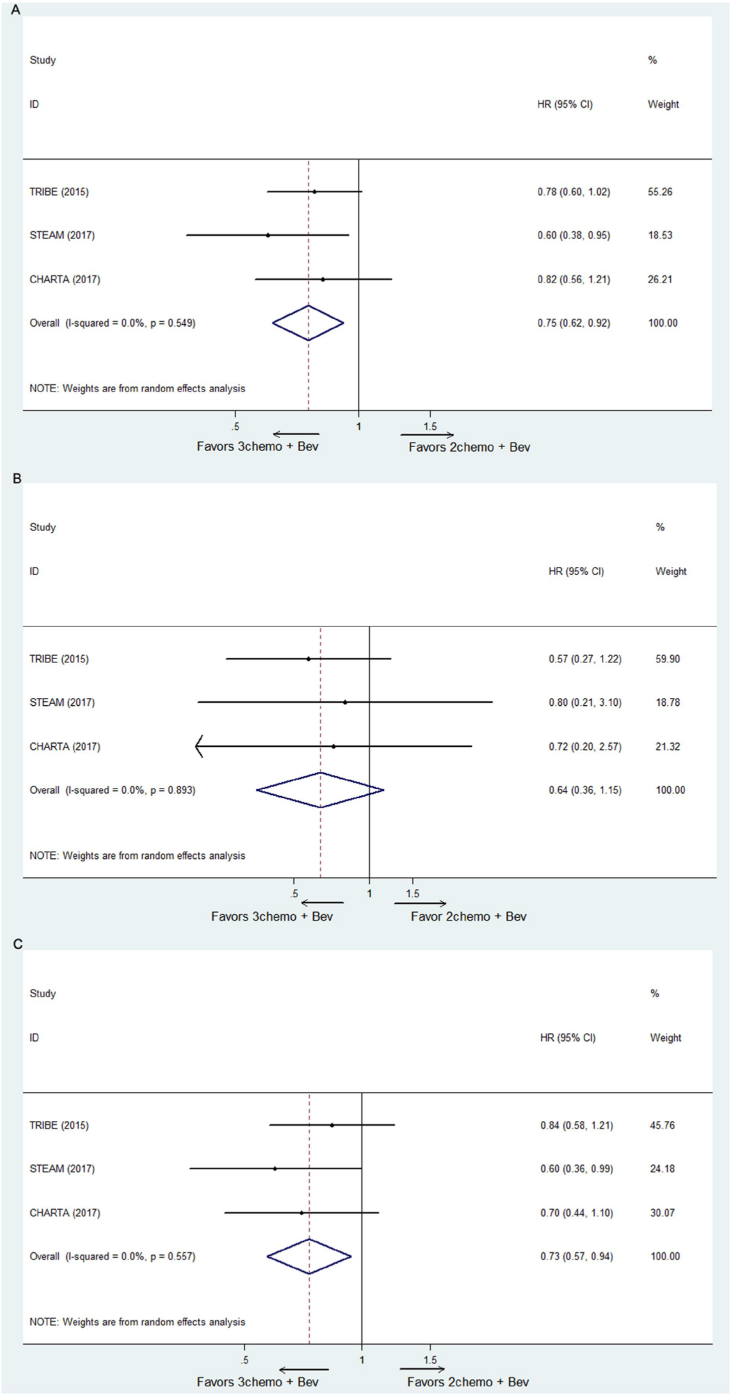

Supplementary Figure 1: Pooled HRs of PFS of the patients with *RAS* mutations **(A)**, with *BRAF* mutations **(B)** and without *RAS* mutations **(C)** using direct meta-analysis. Abbreviations: HR, hazard ratio, PFS, progression-free survival.
